# Photodynamic Therapy 2.0

**DOI:** 10.3390/biomedicines12112425

**Published:** 2024-10-22

**Authors:** Kyungsu Kang, Stefano Bacci

**Affiliations:** 1 Center for Natural Product Systems Biology, Gangneung Institute of Natural Products, Korea Institute of Science and Technology, Gangneung 25451, Gangwon-do, Republic of Korea; kskang@kist.re.kr; 2 Natural Product Applied Science, KIST School, University of Science and Technology (UST), Gangneung 25451, Gangwon-do, Republic of Korea; 3 Research Unit of Histology and Embryology, Department of Biology, University of Florence, Viale Pieraccini 6, 50139 Florence, Italy

In 1903, Von Tappeiner and Jesionek [1] demonstrated the effectiveness of light therapy when combined with a photosensitizer and oxygen—a phenomenon known as “photodynamic action”. Photodynamic therapy (PDT) is widely used in the medical field to treat a variety of oncological and non-oncological human disorders; it generates a high amount of reactive oxygen species that destroy oncological tissue, keratosis, and pathogens. In contrast, recent studies have demonstrated that mild PDT also has beneficial effects, such as wound healing [2], stress and pathogen resistance, and extended lifespan in animal models, such as *Caenorhabditis elegans*, under both normal and pathogen-infected conditions [3,4].

This Editorial, published in *Biomedicines*, provides a current and concise overview of “Photodynamic Therapy 2.0” and discusses recent studies pertaining to PDT. We provide a summary of the various contributions to date, emphasizing novel analytical methods and recent advancements in PDT.

Bowen’s disease is a form of cutaneous squamous cell carcinoma (cSCC) that has a high risk of progression to an invasive form [5]. Conventional PDT is a primary treatment option, with varying protocols involving photosensitizers, light sources, and combinations of the two. Dermoscopy and re-reflectance confocal microscopy can monitor the therapeutic response. Treatment is generally well tolerated, with mild side effects and good cosmetic outcomes. A periodic follow-up is necessary because of the risk of recurrence and progression to cSCC [6]. As the incidence of the keratinocyte tumor increases, the opportunity for PDT will expand [7].

PDT has shown promise in treating brain tumors, particularly gliomas [8], with improved median survival rates and minimal side effects compared with conventional methods, such as surgery, radiotherapy, and chemotherapy. This promising approach may influence future treatments for this challenging and deadly disease, and further studies may lead to significant advancements in treating this tumor type [9].

Nasopharyngeal carcinoma, a malignancy linked to the Epstein–Barr virus (EBV), is associated with 140,000 deaths annually [10]. This study aimed to determine the efficacy of PDT in modulating the tumor microenvironment during nasopharyngeal carcinoma treatment. The review included a search of various databases and used the Oral Health Assessment Tool to assess bias. A meta-analysis revealed that PDT treatment significantly increased Interleukin (IL)-8, IL-1α, IL-1β, Microtubule Associated Proteins 1A/1B chain 3b (LC3) BI, LC3BII, Matrix Metallo Proteinase (MMP) 2, and MMP 9 levels in nasopharyngeal carcinoma cells, while reducing Nuclear Factor Kappa-Light-Chain-Enhancer of Activated B cells (NF-κB), miR BamHI-A Region Rightward Transcript (BART) 1-5p, BART 16, and BART 17-5p levels. PDT also reduced apoptosis and the viability of nasopharyngeal cancer cells infected with EBV. This treatment also increased latent membrane protein 1 levels compared with the control group. Further preclinical studies are needed to validate these results [11].

Furthermore, Fernández-Guarino et al. compared the efficacy of PDT with methyl aminolaevulinic acid (MAL) and aminolaevulinic acid (ALA) in a long-term study of multiple actinic keratosis (AK). A total of 46 patients were treated, including 24 for MAL and 22 for ALA. The data indicated no significant differences at 12 months, despite ALA exhibiting slightly superior results after three months. For PDT, both ALA and MAL were efficacious at lower levels of red light. Long-term efficacy was demonstrated; however, additional studies are needed to determine the lowest point of red light exposure without losing efficacy [12]. Ulrich et al. reported the results of a phase III clinical trial using red light PDT plus a BF-200 ALA (10% 5-aminolevulinic acid nanoemulsion) gel on AK. After 12 weeks of PDT in 21 patients, complete lesion clearance rates were 90.9% with PDT compared with 18.6% in the vehicle control group; the lesion recurrence rate with PDT was 29%. Most patients (81%) rated their satisfaction with PDT as very good or good compared with the vehicle control group (42.8%) [13].

Katalinic et al. examined the effect of photoactivating 3% hydrogen peroxide with a 445 nm diode laser on dental implants infected with *Staphylococcus aureus* and *Candida albicans* biofilms. They assigned 80 contaminated titanium implants to the following four groups: (1) negative control (no therapy); (2) positive control (0.2% chlorhexidine); (3) 3% hydrogen peroxide; and (4) photoactivated 3% hydrogen peroxide. A significant difference was observed between all groups and the negative control, which indicates that the new antimicrobial treatment warrants further study and development [14].

Moreover, Rostami et al. examined the effectiveness of a combination of shockwave-enhanced emission photoacoustic streaming (SWEEPS) and PDT with indocyanine green (ICG) for eliminating the biofilm of *Enterococcus faecalis* from infected root canals. After sterilizing and infecting the canals with *E. faecalis* for two weeks, 30 standardized single-canal teeth from healthy humans were used. The teeth were divided into the following six groups: control, ICG, ICG + 808 nm laser, ICG + SWEEPS, ICG + 808 nm laser + SWEEPS, and sodium hypochlorite (NaOCl, 5.25%). The number of colony-forming units per milliliter (CFUs/mL) was determined for each group. Despite significantly reduced bacterial levels in the ICG-treated group in conjunction with an 808 nm diode laser and SWEEPS, the results indicated that NaOCl alone was the most effective. When comparing ICG with an 808 nm diode laser to ICG with SWEEPS, a statistically significant difference was observed. Based on the results, SWEEPS may effectively increase the dispersion of the photosensitizer in the root canal space, and when combined with an irrigant, it provides promising outcomes [15].

Pordel et al. studied the effects of antimicrobial PDT using a blue diode laser (BDL) with varying output powers and photosensitizers (riboflavin and curcumin) for the treatment of *Streptococcus mutans* around orthodontic brackets. A total of 36 orthodontic brackets were contaminated with *S. mutans* and randomly assigned to the following 12 groups: control, riboflavin alone, riboflavin + different powers (200, 300, 400, or 500 mW) of BDL, curcumin alone, curcumin + different powers (200, 300, 400, or 500 mW) of BDL, and 0.2% chlorhexidine (CHX, positive control). Orthodontic brackets were exposed to BDL at a power density of 0.4–1.0 W/cm^2^ for 30 s, and mean CFUs/mL were measured before and after treatment. The results indicated that CHX and curcumin plus BDL with an output power of 500 mW showed the greatest reduction in *S. mutans* colony numbers, whereas riboflavin alone and riboflavin + BDL had no significant difference in the control group. The study concluded that antimicrobial photodynamic therapy (aPDT) plus curcumin, as a photosensitizer, and BDL successfully suppresses *S. mutans* colonies around stainless steel brackets [16]. Ha et al. examined the effects of aPDT using red light (660 nm) and a new natural photosensitizer, the ethanol extract of *Ligularia fischeri* (LFE), against various pathogenic bacteria. PDT with 20 μg/mL of LFE and red light (120 W/m^2^, 15 min) resulted in a marked antimicrobial activity against methicillin-resistant *S. aureus* (MRSA) as well as *S. mutans*, with a log reduction of 4.7 and 4.9 in viable cells of MRSA and *S. mutans*, respectively. The use of aPDT with LFE showed a much stronger effect compared with vancomycin (100 µg/mL) or ampicillin (100 µg/mL) treatment, with a log reduction of 2.0 and 0.7 in viable cells of MRSA and *S. mutans*, respectively. aPDT decreased the MRSA bacterial cell number in *C. elegans* and extended the lifespan of MRSA-infected worms [17].

Sleep quality is linked to glioma-specific outcomes, including survival [18]. The sleep-induced activation of brain drainage (BD) is important for the survival of glioma patients as it suppresses BD. Photobiomodulation (PBM) is an effective technology for stimulating BD and as an add-on therapy for glioma. A study on male Wistar rats by Shirokow et al. revealed that PBM during sleep stimulates BD more strongly compared with when awake. The study also revealed greater effects of PBM on BD stimulation and the immune response against glioma, including increased CD8+ cells, apoptosis activation, and cell proliferation blockage. This new sleep–phototherapy technology may improve the management of brain cancer patients using smart sleep and non-invasive approaches to glioma treatment [19].

Shakhova et al. examined the efficacy of post-operative phototherapy in mice with CT-26 tumors following excision using a cold knife and a laser scalpel. After the operation, PDT with a photosensitizer based on chlorin was administered at wavelengths of either 405 or 660 nm. The laser scalpel demonstrated superior efficacy compared with the cold knife. The use of PDT following cold knife resection resulted in a decrease in the recurrence rate to 70% and 42% at 405 nm and 660 nm wavelengths, respectively. The use of PDT following laser scalpel resection resulted in recurrence rates of 18% and 30%, respectively. Fluorescence confocal imaging demonstrated that the photosensitizer penetrated more deeply in the cold knife scenario, suggesting a greater effect of PDT at deeper levels. Tumor recurrence was observed in the group exposed to low-dose light without PDT, as evidenced by the disparity in recurrence rates between the 405 nm and 660 nm groups. Light exposure that involved irradiation alone resulted in increased rates of recurrence compared with those administered with PDT. Thus, PDT processing is exclusively advisable for cold knife treatment [20].

In the context of this research, there was an opportunity to meet with professionals in the field of PDT. In terms of content, the reviews that have been recommended are clear and the research articles that have been proposed include innovative techniques and treatment targets that will undoubtedly be further developed in the future. Based on the findings of these studies, it may be concluded that the clinical field is experiencing a greater sense of optimism over the elimination of diseases that have persistently plagued humanity.
